# Observation of Linear Magnetoresistance in MoO_2_

**DOI:** 10.3390/nano14110915

**Published:** 2024-05-23

**Authors:** Yulong Su, Zhibin He, Ruizheng Jiang, Jundong Zhang

**Affiliations:** Marine Engineering College, Dalian Maritime University, Dalian 116026, China; hezb999@dlmu.edu.cn (Z.H.); jrzdmu@dlmu.edu.cn (R.J.); zhjundong@dlmu.edu.cn (J.Z.)

**Keywords:** linear magnetoresistance, MoO_2_, polycrystalline, magneto-transport

## Abstract

Magnetoresistance, the change in resistance with applied magnetic fields, is crucial to the magnetic sensor technology. Linear magnetoresistance has been intensively studied in semimetals and semiconductors. However, the air-stable oxides with a large linear magnetoresistance are highly desirable but remain to be fully explored. In this paper, we report the direct observation of linear magnetoresistance in polycrystalline MoO_2_ without any sign of saturation up to 7 T under 50 K. Interestingly, the linear magnetoresistance reaches as large as 1500% under 7 T at 2 K. The linear field dependence is in great contrast to the parabolic behavior observed in single-crystal MoO_2_, probably due to phonon scattering near the grain boundaries. Our results pave the way to comprehending magneto-transport behavior in oxides and their potential applications in magnetic sensors.

## 1. Introduction

Magnetoresistance refers to the variation in resistivity under the applied magnetic fields. The study of magnetoresistance has been stimulated by the magnetic sensor technology due to its accuracy and non-contact sensing capabilities, thus ensuring a reliable and robust performance even in harsh environmental conditions. The first magnetoresistive effect was discovered in 1856 by William Thomson [1]. Later, Albert Fert and Peter Grünberg discovered giant magnetoresistance in multilayers composed of ferromagnetic and non-magnetic conductive layers and were awarded the 2007 Nobel Prize in Physics, setting up the foundation for the study of spintronics. The magnetic sensors are practically used in magnetic position sensing [2,3], storage/recording devices [4,5,6], non-destructive monitoring [7], biosensing platforms [8,9], and so on. Nowadays, the demand for highly sensitive and cost-effective magnetoresistance sensors has been rising due to the growing concerns about further improving the data storage capacity/detection sensitivity of current magnetoresistance sensors and cutting down the total cost of fabrication/production, which leads to the expansion of the application area of magnetoresistance sensors. Specifically, with emerging nanotechnology, the aim is to develop novel nanomaterials that exhibit large resistance change, with minimum requirements on extreme conditions and large magnetic fields.

Usually, the magnetoresistance exhibits a quadratic dependence in the low magnetic field limit, while under strong magnetic fields, it saturates to a constant value [10], as shown in the following Equation (1):

(1) $$\frac{∆\rho }{\rho }\propto \left\{\begin{array}{cc} {\left(\mu H\right)}^{2}, & \mu H<1\\ C, & \mu H\geq 1 \end{array}\right.$$

where μ is the carriers’ mobility, and *H* is the magnetic field.

This classical magnetoresistance is usually limited to an amplitude of a few percentages. Moreover, researchers found that the amplitude or the magnetic field dependence of the magnetoresistance can be modified by multiple factors, such as magnetic ordering [11,12,13,14], Dirac physics [15], Landau levels [16], or spatial inhomogeneities [17]. In particular, Dirac physics in topological insulators, semimetals, or spatial inhomogeneities can induce pronounced linear magnetoresistance behavior. Recently, linear magnetoresistance under high fields was observed in some Dirac or Weyl semimetals such as Bi [18], InSb [19], Cd_3_As_2_ [20], NbP [21], LaSb [22], and ZrSiS [23]; and compensated semimetals such as WTe_2_ [24,25], TaAs_2_, and NbAs_2_ [26]. There have also been reports of linear magnetoresistance in layered charge density wave and spin density wave compounds, such as iron pnictides [27,28], 2H-NbSe_2_, and 2H-TaSe_2_ [29]. The non-saturating linear magnetoresistance is of great interest for the magnetic sensors with high resolution and memory reading/storage applications. In spite of the intensive study on semimetals and semiconductors, the air-stable oxides with large linear magnetoresistance are highly desirable but remain to be fully explored. In this study, we focused on polycrystalline Molybdenum dioxide (MoO_2_), an air-stable oxide and carried out a systematical study on its magnetic field-dependent resistivity.

MoO_2_, owing to its monoclinic crystal structure (space group ${P2}_{1}/c$), is known as a useful anode material in Li-ion batteries due to its low electrical resistivity, high stability, large capacity, and high density associated with volume capacity [30,31]. Recently, crystalline MoO_2_ was predicted to be a nodal-line semimetal when spin–orbital coupling is neglected [32]. A transport study revealed a quadratic field dependent magnetoresistance in single-crystalline MoO_2_, and the maximum magnetoresistance reaches a value of 5.03 × 10^4^% at 2 K and 9 T [33]. Substantially, two-dimensional MoO_2_ nanoplates were prepared by chemical vapor deposition methods [34,35], which display a large linear magnetoresistance of up to 455% at 3 K and −9 T and a nonlinear Hall effect [35]. Here, we report the visualization of linear magnetoresistance behavior in polycrystalline MoO_2_, in great contrast with the quadratic behavior in single crystals. The polycrystalline MoO_2_ samples were synthesized by a facile technique spark plasma sintering. The linear magnetoresistance behavior is robust, ensuring the practical application. Thus, our experimental results pave the way to a better understanding of the magnetoresistance physics and the potential applications in magnetic sensors.

## 2. Materials and Methods

### 2.1. Synthesis of MoO_2_ Sample

The MoO_2_ samples were prepared by a spark plasma sintering (SPS) method. MoO_2_ powders were grounded for 90 min, using a mortar. Then, the grounded powder was put into a graphite die and sintered by an SPS system (LABOX-650, SINTER LAND, Kanagawa, Japan) at 973 K for 2 h, under vacuum, with a uniaxial pressure of 50 MPa. The sample was subsequently cooled to room temperature naturally. The ingots obtained from sintering were 20.7 mm in diameter and 10 mm in thickness. The obtained pellets were polished and cut into bards with a specific shape and size for electric transport measurements.

### 2.2. Materials Characterizations

The MoO_2_ sample was characterized by the X-ray diffraction (XRD) and scanning electron microscope (SEM) measurements. The XRD patterns were collected in SmartLab, with a Cu-K_α_ radiation source (λ = 1.5406 Å, Rigaku, Tokyo, Japan), with a 2 theta range of 10–80° at room temperature, with a scanning rate of 13.5 degree per min. Scanning electron microscopy (SEM) characterization was performed on an FEI QUANTA 200 FEG microscope (City of Hope, Duarte, CA, USA) at 20 kV.

### 2.3. Transport Measurements

The cleaved samples with a typical size of about 3.0 × 1.0 × 0.1 mm^3^ were used for transport measurements. The resistivity measurements were performed on a Quantum Design physical property measurement system (PPMS), with the highest magnetic field being 7 T. Standard four-probe resistivity (*ρ*_xx_) measurements were carried out using a constant current mode. The magnetoresistance (MR) is defined as MR = [*ρ_xx_*(*H*, *T*) − *ρ_xx_*(0, *T*)]/*ρ_xx_*(0, *T*) and measured by applying the magnetic field, *H*, perpendicular to the sample. Electrical contacts were prepared by platinum wires and a silver paste. The Seebeck coefficient (*S*) was measured by the PPMS thermal transport option in continuous scanning mode, with a 0.2 K min^−1^ heating rate.

## 3. Results and Discussion

### 3.1. XRD and SEM Analysis of MoO_2_ Samples

**Figure 1:**
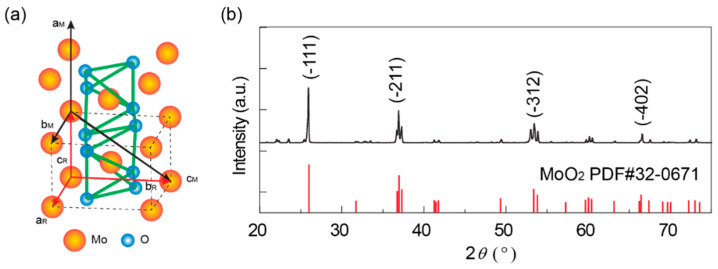
Crystal structure of MoO_2_. (**a**) Schematic ball-and-stick model of monoclinic (M) MoO_2_. The unit cell of the distorted rutile (R) structure is shown with dashed lines. (**b**) Powder XRD pattern of MoO_2_.

**Figure 2:**
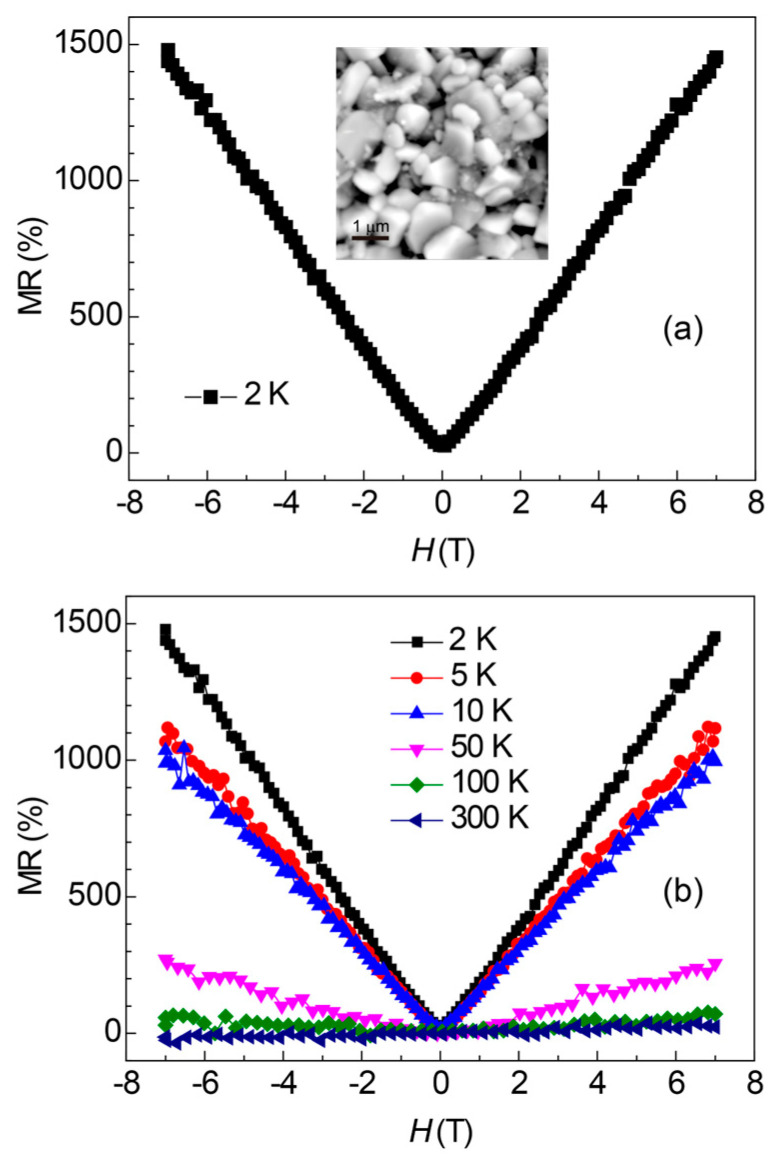
Magnetoresistance (MR) of polycrystalline MoO_2_. (**a**) MR at 2 K. Here, MR is defined as MR = [*ρ_xx_*(*H*, *T*) − *ρ_xx_*(0, *T*)]/*ρ_xx_*(0, *T*) × 100%. Inset: Scanning electron microscopy image of polycrystalline MoO_2_. (**b**) MR at various temperatures ranging from 2 K to 300 K.

Figure 1a displays the schematic crystal structure of monoclinic MoO_2_, which can also be considered to have a distorted rutile structure. Figure 1b shows the X-ray diffraction pattern of the prepared polycrystalline MoO_2_ sample. All the characteristic peaks of the XRD pattern can be indexed into well-crystallized MoO_2_, confirming its monoclinic crystal structure (PDF#32-0671) with the space group, ${P2}_{1}/c$; and lattice constants, *a* = 5.69 Å, *b* = 3.12 Å, *c* = 5.07 Å, and *β* = 112.78°. The grain size of MoO_2_ was examined by the SEM in Supplementary Figure S1 and inset of Figure 2a, where the grain size ranges from 500 nm to 1.0 μm.

### 3.2. Magneto-Transport Properties of MoO_2_ Samples

Figure 2 shows the temperature-dependent magnetoresistance by applying magnetic fields perpendicular to the sample. A positive, linear magnetic field dependence of the transverse magnetoresistance is visualized up to 7 T in Figure 2a. It is more obvious in the first-order derivative, dMR(*H*)/d*H* (Supplementary Figure S2), that the linear dependence appears from 0.1 T. It is worth mentioning that the amplitude of the magnetoresistance is huge, reaching 1500% at *H* = 7 T and *T* = 2 K. Moreover, varying the temperature, *T*, from 2 K to 50 K dramatically affects the magnitude of MR but changes neither the sign nor the shape of MR-*H* curves, as shown in Figure 2b. It is obvious that the linear MR is strongly dependent on the temperature, *T*, and barely exists at a *T* above 50 K. The sensitivity of the magnitude of *MR* with *T* suggests the significant role of phonon scattering, which leads to a remarkable decrease with the increasing *T*. We need to mention that the linear magnetoresistance behavior is air-stable in polycrystalline MoO_2_ and barely changes within three months.

**Figure 3:**
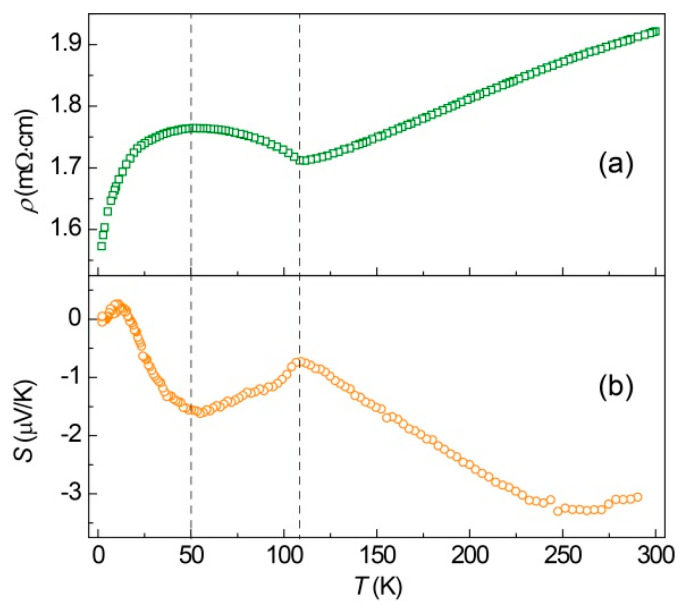
Temperature-dependent resistivity *ρ*(*T*) (**a**) and Seebeck coefficient *S*(*T*) (**b**) of polycrystalline MoO_2_.

To shed more light on the linear magnetoresistance behavior, we carried out a thermal transport measurement for the MoO_2_ samples. Figure 3 displays the temperature-dependent resistivity, *ρ*(*T*), and Seebeck coefficient, *S*(*T*), curves. As shown in Figure 3a, the *ρ*-*T* curve of MoO_2_ exhibits a metallic behavior. Above 100 K, the resistivity, *ρ*, increases with the increase in the temperature, *T*, while below 100 K, the resistivity, *ρ,* first increases and then decreases with a turnover at *T* = 50 K. It is different with single-crystal data of MoO_2_, where the decrease in *ρ* below 50 K is absent probably due to electron scattering at the grain boundaries [33]. Figure 3b displays the temperature-dependent Seebeck coefficient, *S,* below 300 K. The Seebeck coefficient exhibits a non-monotopic temperature dependence, with the sign of the Seebeck coefficient changing from negative to positive with decreasing temperature. The negative Seebeck coefficient, *S*, below 300 K indicates electrons as the charge carrier in this system. However, there is a sign change from 16.7 K to 2 K and peaks at 10.8 K with a positive Seebeck coefficient.

The Seebeck coefficient, *S*, can be described within a free electron model by Equation (2):

(2) $$S\left(T\right)=-\frac{\pi^{2}}{3}\frac{k_{B}}{\left|e\right|}k_{B}T\left[\frac{N(0)}{n}+\frac{1}{\mu }\frac{d\mu }{dE_{F}}\right]$$

where $E_{F}$ is the Fermi energy, and N(0) is the density of states. The first term, *n*, determines the carrier type, while the second one can be qualitatively related to the variation in mobility and may change signs with temperature.

The Seebeck coefficient can also be calculated by Equation (3):

(3) $$S=\frac{S_{e}b+S_{h}}{b+1}$$

where *S* is the total Seebeck coefficient; $S_{e}$ and *S_h_* are the Seebeck coefficient of electrons and holes, respectively; and $b=\mu_{e}/\mu_{h}$ is the ratio of the mobilities of electrons and holes. It is known that the mobilities strongly depend on the temperature, as well as the crystallography, such as orientation and defects. Assuming identical charge carrier concentrations for holes and electrons, the sign change in the Seebeck coefficient happens when the mobility of holes becomes larger than the mobility of the electrons, which can occur because the mobility of electrons and holes is affected differently by surface scattering due to their different effective masses [36]. Similar explanations were given for the sign change in Bi, which suggested that the electron mobility becomes smaller than the hole mobility in the low temperature range, because the hole mobility was not significantly affected by boundary scattering [37].

Next, we discuss the possible origins for the linear magnetoresistance behavior, which is different from the parabolic magnetic field dependence in the MoO_2_ single crystal. First, the observed quadratic magnetic field dependence in single-crystalline MoO_2_ [33] can get out of the possible origin from Dirac physics, even though MoO_2_ is predicted to be a nodal-line semimetal [34]. Moreover, the absence of linear magnetoresistance in the single crystal can also remove the possibility of magnetic ordering or Landau levels. Last but not least, non-saturating linear magnetoresistance can be comprehended semi-classically in the Parish and Littlewood framework [18] by a random resistor network model that mimics a disordered and strongly inhomogeneous conductor system. It has been evidenced that voids or inhomogeneities will cause linear magnetoresistance in disordered and strongly inhomogeneous semiconductors such as InSb [20]. In our polycrystalline MoO_2_ samples, the grain size is of 0.5–1.0 μm, and the crystalline imperfections, especially grain boundaries, cannot be ignored. Thus, enhanced phonon scattering is expected near the grain boundaries [38,39]. We notice that the chemical vapor deposition-grown MoO_2_ nanoplates (with a lateral size of ~10 μm and a thickness of 7.8–55.2 nm) also display a large linear magnetoresistance of up to 455% at 3 K and −9 T [35]. In two-dimensional films, the phonon–interface or phonon–boundary scattering increases as the film becomes thinner [40,41], especially when the sample size along the transport direction is much smaller than the mean free path of the phonon. Even though the grain boundaries are absent in those MoO_2_ nanoplates [35], phonon–interface scattering is inevitable since the thickness range of 7.8–55.2 nm is considerably smaller than the phonon mean free path of the phonon, i.e., ~300 nm in silicon films [41]. Therefore, we believe that the grain boundary plays a remarkable role in the linear magnetoresistance behavior in polycrystal MoO_2_, which is consistent with the sensitive temperature dependence, where the linear magnetoresistance disappears as temperature increases above 50 K (Figure 2b). More theoretical or experimental work in the future is needed to clarify the physical origin of this linear magnetoresistance in polycrystalline MoO_2_.

## 4. Conclusions

In this paper, we report a linear magnetoresistance behavior in polycrystalline MoO_2_ prepared by the spark plasma sintering technique. The linear magnetoresistance does not saturate up to 7 T under 50 K, with the measured magnitude reaching as large as ~1500% at *T* = 2 K and *H* = 7 T. Polycrystalline MoO_2_ is air-stable and, thus, ensures a reliable and robust magnetoresistance performance even in harsh environmental conditions. Moreover, the facile synthesis of polycrystalline MoO_2_, together with the feature of robust linear magnetoresistance, guarantees mass production and practical application. Our results provide valuable guidance towards the comprehension of magneto-transport behavior in oxides, as well as their potential applications in magnetic sensors.

## Data Availability

Data will be made available upon request.

## Supplementary Materials

The following supporting information can be downloaded at https://www.mdpi.com/article/10.3390/nano14110915/s1, Figure S1. SEM images of polycrystalline MoO_2_ samples; Figure S2. The magnetic field derivatives of MR at 2 K, 5 K, 10 K, and 50 K; Figure S3. R-T curves under 0 T and 7 T.
