# Supplementary material for: Observation of Linear Magnetoresistance in MoO2
Source: Nanomaterials (Basel). 2024 May 23;14(11):915. doi: 10.3390/nano14110915 (PMC11173525; doi:10.3390/nano14110915)
Supplement: Supplementary file 1 [file nanomaterials-14-00915-s001.zip › nanomaterials-2981316-supplementary.pdf]

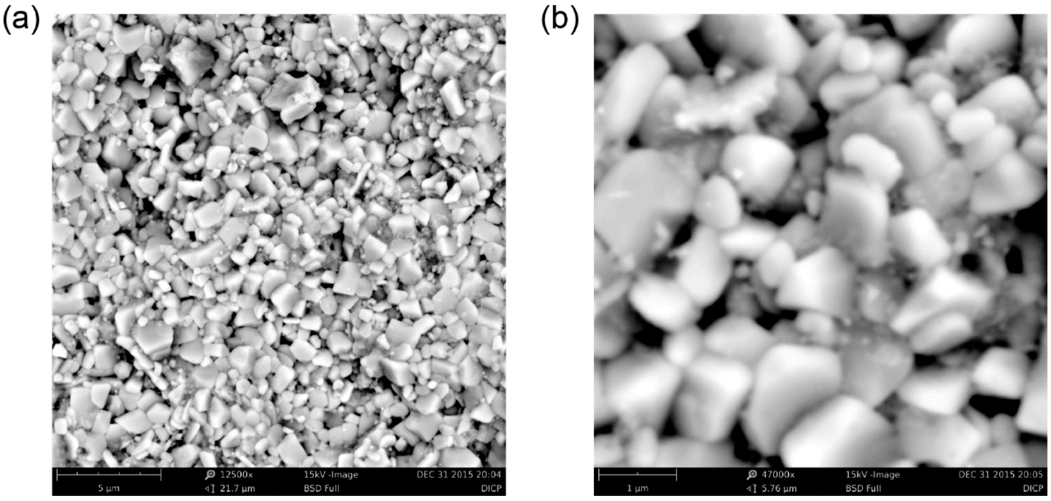

Figure S1. SEM images of polycrystalline MoO_2. The SEM image were taken by a scanning electron microscopy (FEI Quanta 200 FEG).

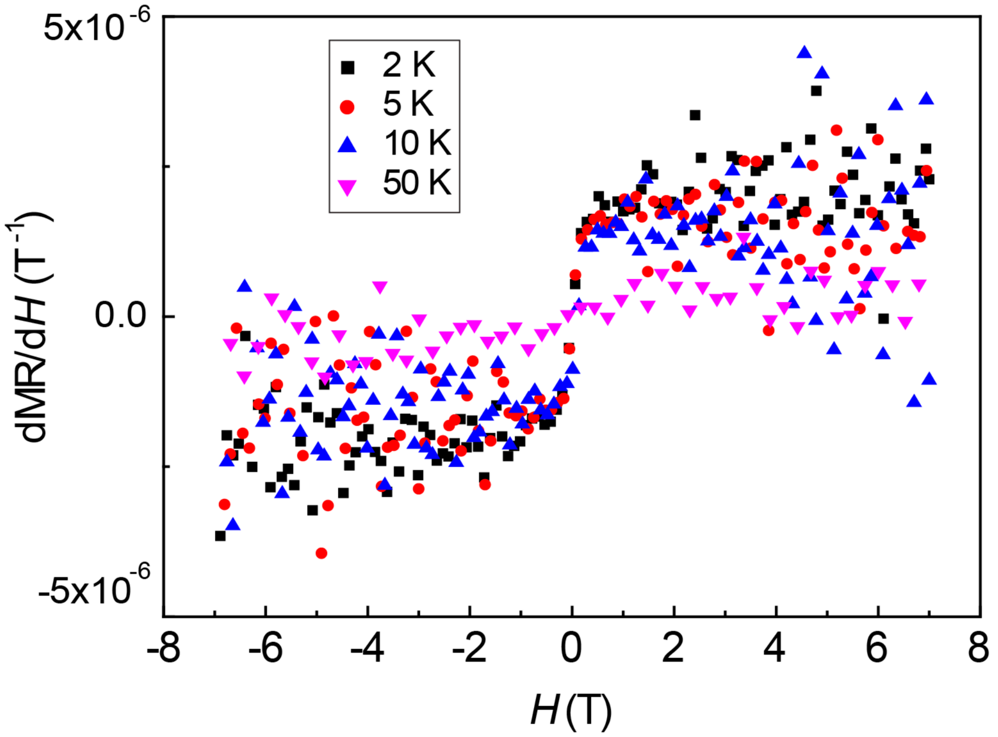

Figure S2. The magnetic field derivatives of MR at 2 K, 5 K, 10 K and 50 K.

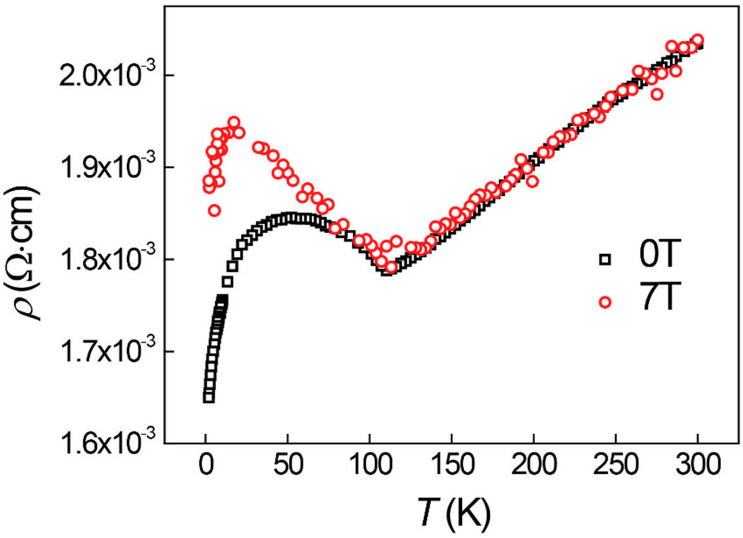

Figure S3. Resistivity ρ (T) of polycrystalline MoO_2 as a function of temperature measured under H = 0 T and 7 T.
